# Antioxidant Effect of Thioredoxin and Vitamin D3 in Peritoneal Dialysis Patients

**DOI:** 10.1155/2022/2590944

**Published:** 2022-05-02

**Authors:** Sara Yavuz Ileri, Adnan Batman, Ceyla Eraldemir, Serkan Bakırdogen, Erkan Dervisoglu

**Affiliations:** ^1^Department of Nephrology, Ankara Dr. Abdurrahman Yurtaslan Oncolology Training and Research Hospital, Ankara, Turkey; ^2^Department of Endocrinology, Koc University School of Medicine, Istanbul, Turkey; ^3^Department of Biochemistry, Kocaeli University Medical Faculty, Kocaeli, Turkey; ^4^Department of Nephrology, Canakkale University School of Medicine, Canakkale, Turkey; ^5^Department of Internal Medicine Nephrology Division, Kocaeli University Medical Faculty, Kocaeli, Turkey

## Abstract

**Background:**

Among the chronic diseases, chronic kidney failure is one of diseases that have the most difficulty in coping with oxidative stress due to the deterioration of the antioxidant system balance in the body. Beyond being a vitamin, 1*α*,25-dihydroxycholecalciferol (vitamin D3) is a molecule that positively or negatively affects many enzymes which are in protein structures. Thioredoxin (TRX), which has an important role in the antioxidant system, is one of these proteins. By conducting this study, we wanted to emphasize the role of vitamin D3 in reducing the oxidative stress load on patients undergoing peritoneal dialysis (PD) via serum TRX level measurement.

**Methods:**

In this study, we evaluated the medical treatments of 69 PD patients who were followed up routinely. The patients were divided into 2 groups according to whether they used vitamin D3 or not. 49 of our patients were using vitamin D3. While requesting routine laboratory tests, we reserved a separate serum sample to measure serum TRX levels by double-antibody sandwich enzyme-linked immunosorbent assay for all patients.

**Results:**

Only one parameter has a significant statistical relationship with serum TRX level and the treatment protocol. The serum TRX level was significantly higher (211,62 U/l ± 314,46) in the group receiving vitamin D3 compared to the group which is not using Vitamin D3 (101,63 U/l ± 215,03) (*p* < 0,006).

**Conclusion:**

This study highlights the importance of appropriate dose of vitamin D3 replacement especially in PD patients who are under intense oxidative stress compared to healthy individuals.

## 1. Introduction

Thioredoxin (TRX) is a small 12 kDa protein that plays a cytoprotective role against many oxidative stresses in various systems. It is one of the constituent elements of the important protective system against oxidative stress. The components of this system include TXN, thioredoxin-interacting protein (TXNIP), thioredoxin reductase, and nicotinamide adenine dinucleotide phosphate (NADPH). It was first purified and described as being the hydrogen donor for ribonucleotide reductase (RNR) in Escherichia coli in 1964 [1]. TRX plays a role in many physiological cellular responses both inside and outside the cell. It reduces oxidative stress by collecting reactive oxygen radicals inside the cell and acts as a growth factor outside the cell, triggering cell growth. Its most important feature is the reduction of free radicals. It also protects cells from TNF, hydrogen peroxide, activated neutrophils, and ischemic reperfusion injury. In a healthy body, oxygen radicals and antioxidant defense mechanisms work in perfect balance. The situation that occurs when this balance is disrupted in favor of radicals is called oxidative stress. Oxidative stress was first explained by Seis in 1985 as the damage to tissues by failing to maintain the balance between insufficient antioxidant defense mechanism of cells and excessively produced free oxygen radicals [2].

Chronic kidney disease (CKD) is a common and serious problem that adversely affects human health, limits longevity, and increases costs to health-care systems worldwide. It has an increasing incidence and prevalence in developed and developing nations. Globally, around three million patients are currently receiving renal replacement therapy (RRT) and this number is expected to increase to between 5 and 10 million by 2030 [3]. Its increasing incidence cannot be fully explained by traditional risk factors. Oxidative stress is prevalent in CKD patients and is an important pathogenic mechanism [3]. Because the kidney is an organ with a rapid metabolism where mitochondrial oxidation-reduction reactions are quite intense, increased reactive oxygen radicals together with the depletion of antioxidant molecules in chronic kidney patients cause the kidney to become more resistant to this oxidative stress. The inability to eliminate this oxidative stress accelerates the progression of chronic kidney disease and shortens the path to end-stage kidney disease. In fact, this process is much more aggressive in diabetic patients [4]. In addition, increased oxidative stress increases the development of complications such as anemia, inflammation, atherosclerosis, and hypertension in chronic kidney patients [5]. A lot of studies have shown a significant imbalance in prooxidant and antioxidant activities in patients with renal dysfunction [6]. Therefore, antioxidant system has a lot of important pathophysiological roles in those patients.

Another molecule associated with vitamin D that fights oxidative stress is Klotho (Klt). Klt is discovered by Kuro-o et al. in 1997. It is mainly expressed in 2 locations in the human body: the kidneys and brain. Klt has antioxidant, anti-inflammatory, and tumor suppressive features; in addition, Klt is required to bind FGF-23 to the FGF receptor and to activate intracellular signal molecules in the regulation of P and vitamin D metabolism. FGF-23 inhibits 1*α*-hydroxylase and upregulates 24*α*-hydroxylase, so vitamin D 3 levels decrease [7].

Vitamin E, another antioxidant, has already been shown to improve renal anemia and the requirement of erythropoietin supplement in dialysis patients [8], and another study has shown similar findings of improvement in hematocrit with intravenous vitamin C, another antioxidant, and reduced the need for erythropoietin [9]. For this reason, it is important to cope with oxidative stress and to increase the effectiveness of antioxidant mechanisms especially in patients with chronic kidney disease during the progression to end-stage renal disease and RRT. In this study, we aim to contribute to the literature by evaluating the effect of the routine medical treatment, especially vitamin D3 replacement of our 69 PD patients on oxidative stress via TRX system which plays an important role in oxidation and reduction reactions.

## 2. Materials and Methods

The study was conducted with a total of 69 end-stage renal disease patients with estimated glomerular filtration rate (eGFR) < 15 ml/mn according MDRD formula. 50.7% (*n* = 35) were female and 49.3% (*n* = 34) were male. The inclusion criteria were being between the ages of 18 and 65, having regular monthly outpatient follow-ups for at least 2 years and using only biocompatible PD solutions. 62 of our patients were doing continuous ambulatory peritoneal dialysis (CAPD), and 7 of them were doing automated peritoneal dialysis (APD). When we evaluate in terms of CKD etiologies, there were hypertension (HT) in 32 patients, diabetes mellitus (DM) in 19 patients, polycystic kidney disease (PCKD) in 8 patients, glomerulonephritis (GMN) in 2 patients, and other causes in the rest.

The exclusion criteria were having bacterial peritonitis within one month, any another previous (within one week), or acute infection and unexplained serum C-reactive protein (CRP). We divided our patients into 2 groups according to vitamin D3 use. From retrospective analyses, we determined that 49 out of 69 patients were already under the vitamin D3 supplementation at least 3 months but 20 out of 69 were not eligible due to their serum iPTH, Ca, P levels.

Apart from routine outpatient follow-up laboratory parameters which were routinely measured with the kits studied in the biochemistry laboratory of our university, we also measured serum TRX levels and vitamin D3 levels for all patients. TRX is measured by double-antibody sandwich enzyme-linked immunosorbent assay (ELISA) kit (Hangzhou East BioPharma Co., ltd. China). The lower detection limit for TRX was 2.49 U/l, and the standard range was 5-1500 U/l according to the manufacturer's instruction. Venous blood samples were taken in the morning's fasting state. After at least 30 min, but within 2 h, the tubes were centrifuged at 20°C for 15 min and the sera were stored frozen in plastic vials at -80° until the time of consecutive analyses.

### 2.1. Statistical Reviews

NCSS (Number Cruncher Statistical System) 2007 (Kaysville, Utah, USA) program was used for statistical analysis. Student's *t* test was used for the comparison of normally distributed parameters in comparison of quantitative data as well as descriptive statistical methods (mean, standard deviation, median, frequency, and ratio) when evaluating study data; Kruskal-Wallis test was used in the comparison of the variables between etiology and thioredoxin in Table 1. Mann–Whitney *U* test was used in the determination of the group causing the difference and in the evaluations of the two groups. Spearman's correlation analysis was used to evaluate the relationships between variables. Chi-square test, Yate's Continuity Correction, and Fisher's Exact and Fisher Freeman Halton test were used to compare qualitative data. The results were evaluated at the 95% confidence interval, and significance level of *p* < 0.05.

## 3. Results

The clinical data and characteristics of study patients (*n* = 69) are displayed in Table 2. Most of the patients were 88.4% (*n* = 61) married and 60.8% (*n* = 42) were primary school graduates. In terms of income level, 84.0% (*n* = 58) of them had a medium income level. Body mass index levels ranged from 20.76 to 46.12 with a mean of 29.548 ± 6.0 kg/m^2^. When evaluated in terms of smoking, which is an important increaser of oxidative stress, 11.6% (*n* = 8) of our patients smoked, 36.2% (*n* = 25) did not smoke, and 49.3% (*n* = 34) had never smoked. 87.0% (*n* = 60) had no family history of chronic kidney disease, while 13.0% (*n* = 9) had a family history. When the chronic kidney disease of our patients was evaluated in terms of etiology, 46.4% (*n* = 32) were found to be secondary to hypertension. All our patients were using biocompatible dialysis solution with dextrose (*n* = 69). 58.0% of the group (*n* = 40) were using 1*α*,25-dihydroxycholecalciferol (Vit D3) orally (Table 1). According to comorbidities, 94.1% of the patients (*n* = 64) had hypertension, 29.0% (*n* = 20) had diabetes, 23.5% (*n* = 16) had coronary artery disease, 23.2% (*n* = 16) had heart valve disease, 22.1% (*n* = 6) had chronic heart failure, 14.5% (*n* = 10) had myocardial infarction, and 8.8% (*n* = 6) had chronic obstructive disease (COPD). The mean serum thioredoxin level was 165.39 ± 280.62 U/l. Peritonitis developed in only 2 patients during our follow-up. These two patients who developed peritonitis were in the group that did not use vitamin D3.

We did not find any significant difference between thioredoxin measurements according to PD type, PD solution such as icodextran and amino acid solutions and disease etiology of our patients (*p* > 0.05) (Table 3). We found that the thioredoxin measurements of the cases who routinely use vitamin D3 in their treatment plan were higher than those who did not (*p* < 0.01). We have evaluated in terms of comorbid diseases; we did not find any significant relationship (*p* > 0.05), but the serum thioredoxin level was lower in the COPD group (*p* < 0.01) (Table 4). While there was any meaningful relationship between biochemical values especially inflammatory parameters, such as CRP level, phosphorus, albumin, and thioredoxin measurements (*p* > 0.05), there was positive and statistically significant correlation with serum iron levels (*r* = 0.247; *p* < 0.05) (Table 5).

## 4. Discussion

Chronic kidney patients are more sensitive to oxidative stress due to decreased kidney function, and the process leading to end-stage renal failure of these patients is getting shorter due to continuous exposure and insufficiency of the antioxidant system. Increased oxidative stress occurs even in early stages of the disease, progresses with deterioration of renal function, and is further aggravated by hemodialysis (HD) due to the biocompatibility of the method. Compared to HD, PD is a more biocompatible dialysis modality, but even less high oxidative stress status persists. And unfortunately, this oxidative stress causes loss of residual renal function. Moreover, oxidative stress is linked with peritonitis, inflammation, atherosclerosis, and cardiovascular disease (CVD) and increases mortality in this group which is more prone to coronary artery disease than the general population. Therefore, to ameliorate this status in PD patients is important [10]. In this study, we measured the serum TRX levels of our patients to evaluate the oxidative stress who were followed up continuously in our outpatient peritoneal dialysis clinic. As it is known, TRX is a very important oxidative stress fighter protein. In fact, in a study conducted on transgenic mice, it was determined that over expression of thioredoxin protein prolongs lifespan [11]. It is emphasized that TRX level may have important effects on the pathogenesis of many chronic diseases (including CVD, heart failure, stroke, metabolic syndrome, neurodegenerative diseases, arthritis, and cancer) [12]. When we compared the PD types and PD durations of our patients included in the study in terms of TRN serum levels, there was no significant difference. Our patients who had an average of 836 ml of residual urine were receiving effective renal replacement therapy with 1.7 peritoneal KT/V values and did not have any acute infection, and serum CRP values were within normal limits. We did not find a significant relationship between comorbid diseases and TRX in our patients except for COPD. Serum TRX level was low in COPD patients. We can explain this situation by the lung tissue protection of the TRX from oxidative damage. It has been shown in recent studies that TRX deficiency triggers asthma as well as chronic obstructive pulmonary disease, and increased serum thioredoxin levels have reduced lung tissue damage. It is emphasized that TRX is important for future therapeutic treatments in many pulmonary diseases [13]. It is probably for this reason that we found low TRX levels in the group who had comorbid diseases such as chronic obstructive pulmonary disease.

As we know, mammalian TRX system, which is composed of TRX, TRX reductase, and NADPH, has many biological functions. Besides its antioxidant role by reducing reactive oxygen species (ROS), its classic function is to act as a hydrogen donor for ribonucleotide reductase for DNA synthesis. Another function is to stimulate the proliferation of lymphoid cells and a variety of human solid tumor cell lines. Another key mechanism by which TRX mediates cell protection is via binding to signaling molecules and modulating their function. One of these proteins is thioredoxin-interacting protein (TXNIP). The thioredoxin-interacting protein (TXNIP) promotes oxidative stress by inactivating thioredoxin gene. (TXN). This protein is involved in diverse disease processes including insulin resistance, atherosclerosis, and carcinogenesis. In the body, TXN and TXNIP have also independent effects from oxidative stress. By binding to TXN, TXNIP precludes the apoptosis signal regulation of TRX, triggers apoptosis, and stops the cell cycle. Thus, cell proliferation decreases and apoptosis increases [14]. Thioredoxin-interacting protein expression can be induced by a variety of environmental stimuli including oxidative stress, high glucose, and the administration of vitamin D3 very importantly [15]. They also demonstrated that the other binding protein VDUP1 expression which inhibits the reducing activity of TRX via interacting catalytic active center can be stimulated and decreases thioredoxin antiapoptotic and proliferative function [16].

In our study, when we compared serum TRX levels in terms of the routine treatment protocols, we found them to be higher in the group who received vitamin D3 at varying doses orally (0.5 mcg-3 mcg) because of their serum iPTH, Ca, P values at least 3 months.

We think that the reason for the high serum TRX levels in the patient group using vitamin D3 is probably the presence of negative feedback effect which occurs with the inhibition of TRX activity by VDPU1 and TRNIP. Yet, there is no study in literature on the contribution of vitamin D3 directly to the TRX serum level. However, we did not compare the two groups in terms of the PD solution they used, their serum albumin levels, or their CRP value distributions, and unfortunately, our sample size is quite small. These are our main limitations.

Therefore, further studies are required to make a definitive statement. We can nevertheless state that due to the direct or indirect effects of vitamin D3 in many diseases, excluding bone-mineral disorders, the correct and effective use of vitamin D3 is important in end-stage renal disease patients. The effect is more pronounced in cases where chronic inflammation is active and comorbid factors are more frequent and more destructive. For this reason, whether it is an inpatient or an outpatient, all chronic kidney patients under RRT, should be followed seriously by clinicians for appropriate replacement of vitamin D3 with close monitoring of the serum level of iPTH, Ca, P.

## Figures and Tables

**Table 1:** Distribution of disease-related features.

		min-max	Average ± SD
Peritoneal dialysis duration (year)		0,16-13,11	4, 04 ± 2, 92
CAPD duration		2-156	48, 41 ± 35, 45
Previous HD duration(*n* = 8) (month)		1-144	28, 87 ± 49, 11
Daily dwell volume		6000-13000	8739, 13 ± 1657, 44
Daily dwell changing number		1-5	4, 01 ± 0, 73
Utrafiltration (ml)		0,20-4,0	1, 46 ± 0, 75
Residual urine (ml)		0-3000	827,53 ± 693,59
		*N*	%
Previous hemodialysis	Present	8	11,6
	Absent	61	88,4
Peritoneal dialysis type	CAPD	62	89,9
	APD	7	10,1
Etiology	HT	32	46,4
	DM	19	27,5
	Polycystic kidney disease	8	11,6
	GMN	8	11,6
	Other	2	2,9
Medical treatment	Dextroz sol.	69	100
	Icodextran sol.	36	52,2
	Amino acid sol.	14	20,3
	Erythropoietin	33	47,8
	Blood transfusion	1	1,4
	Antilipidemic	19	27,5
	OAD	1	1,4
	Insulin	18	26,1
	Iron replacement	16	23,2
	Amino acid replacement	28	40,6
	Phosphorus binding	39	56,5
	Vitamin D3	40	58,0
	Other drugs	2	2,8

CAPD: Continuous ambulatory peritoneal dialysis; HD: Hemodialysis; APD: Automated peritoneal dialysis; HT: Hypertension; DM: Diabetes mellitus, GMN: Glomerulonephritis; OAD: Oral antidiabetics.

**Table 2:** Patient characteristics (*n* = 69).

	min-max	Average ± SD
Age (year)	27-82	51, 43 ± 2, 92
Weight (kg)	51-108	77, 79 ± 14, 60
Length (cm)	143-179	162,55 ± 8, 19
BMI (kg/m^2^)	20,76-46,12	29,548 ± 6, 00
Systolic BP (mmHg)	110-200	151,45 ± 22, 57
Diastolic BP (mmHg)	70-110	87, 24 ± 10, 27

**Table 3:** Evaluation according PD type, etiology, and medical treatment.

		Thioredoxin	*p*
		Average ± SD	
PD type	CAPD (*n* = 62)	167,96 ± 285,92 (58,5)	^b^0,921
	APD (*n* = 7)	142,68 ± 246,32 (51,4)	
Etiology	HT (*n* = 32)	101,22 ± 191,7 (54,4)	^c^0,488
	DM (*n* = 19)	298,4 ± 378,89 (69,2)	
	Polycystic kidney disease (*n* = 8)	53, 16 ± 20, 86 (50,1)	
	Others (*n* = 8)	189,1 ± 360,39 (61,5)	
	GMN (*n* = 2)	282,75 ± 344,35 (282,8)	
Drugs			
Icodextran	Absent (*n* = 33)	85, 86 ± 126,93 (50,5)	^b^0,364
	Present (*n* = 36)	238,3 ± 356,13 (65,3)	
Amino acid solutions	Absent (*n* = 55)	127,71 ± 236,3 (53,4)	^b^0,561
	Present (*n* = 14)	313,43 ± 387,76 (68,7)	
Erythropoietin	Not using (*n* = 36)	180,8 ± 312,27 (50,9)	^b^0,576
	Using (*n* = 33)	148,59 ± 245,2 (62,2)	
Oral	Not using (*n* = 50)	149,86 ± 269,99 (52,5)	^b^0,493
Antilipidemic	Using (*n* = 19)	206,28 ± 310,83 (69,2)	
Insulin treatment	Absent (*n* = 51)	124,23 ± 225,93 (51,6)	^b^0,280
	Present (*n* = 18)	282,03 ± 381,26 (73,1)	
Iron treatment	Absent (*n* = 53)	148,52 ± 251,34 (53,4)	^b^0,634
	Present (*n* = 16)	221,3 ± 365,58 (62,4)	
Amino acids	Absent (*n* = 41)	138,24 ± 243,99 (53,4)	^b^0,513
	Present (*n* = 28)	205,16 ± 327,67 (65)	
Phosphorus	Absent (*n* = 30)	160,01 ± 286,3 (53,4)	^b^0,694
Binding	Present (*n* = 39)	169,53 ± 279,86 (54,2)	
Vitamin D3	Absent (*n* = 29)	101,63 ± 215,03 (42,5)	^b^0,006^∗∗^
	Present (*n* = 40)	211,62 ± 314,46 (68,3)	

^b^Mann–Whitney *U* test. ^c^Kruskal-Wallis test. ^∗∗^*p* < 0, 01. CAPD: Continuous ambulatory peritoneal dialysis; APD: Automated peritoneal dialysis; HT: Hypertension; DM: Diabetes mellitus, GMN: Glomerulonephritis.

**Table 4:** Thioredoxin evaluations according to comorbid diseases.

Comorbid disease		Thioredoxin	*p*
		Average ± SD	
Diabetes mellitus	Absent (*n* = 48)	129,08 ± 232,01 (51,5)	^b^0,427
	Present (*n* = 20)	258,06 ± 368,32 (73,1)	
Chronic heart failure	Absent (*n* = 62)	178,68 ± 293,02 (58,5)	^b^0,066
	Present (*n* = 6)	46, 5 ± 43, 06 (36,5)	
Heart valves disease	Absent (*n* = 52)	188,19 ± 308,09 (53,8)	^b^0,623
	Present (*n* = 16)	98, 18 ± 163,81 (55)	
Myocardial infarcts	Absent (*n* = 58)	176,27 ± 293,63 (56,3)	^b^0,146
	Present (*n* = 10)	113,31 ± 209,28 (40,8)	
Chronic obstructive lung disease	Absent (*n* = 62)	180,51 ± 292,33 (60,3)	^b^0,006^∗∗^
	Present (*n* = 6)	27, 56 ± 18, 49 (34,4)	
Hypertension	Absent (*n* = 3)	74, 89 ± 22, 7 (78,8)	^b^0,378
	Present (*n* = 65)	171,27 ± 288,17 (53,4)	
Angina pectoris	Absent (*n* = 65)	172,99 ± 287,44 (54,2)	^b^0,303
	Present (*n* = 3)	37, 5 ± 30, 46 (51,4)	
Coronary artery bypass surgery	Absent (*n* = 62)	162,86 ± 280,99 (51,5)	^b^0,141
	Present (*n* = 6)	209,9 ± 320,58 (85,8)	
Coronary artery disease	Absent (*n* = 52)	174,96 ± 292,58 (56,3)	^b^0,347
	Present (*n* = 16)	141,2 ± 253,33 (48,1)	
Blindness	Absent (*n* = 53)	170,72 ± 290,26 (53,4)	^b^0,912
	Present (*n* = 15)	153,91 ± 261,6 (58,5)	
Infections (including peritonitis)	Absent (*n* = 66)	169,77 ± 286,23 (52,5)	-
	Present (*n* = 2)	75, 96 ± 4, 08 (76)	

^b^Mann–Whitney *U* test. ^∗∗^*p* < 0.01. -: It was not included in the comparison because the number of people was insufficient.

**Table 5:** Relation between thioredoxin and laboratory findings.

	Thioredoxin	
	*r*	*p*
Hemoglobin(gr/dl)	-0,022	0,860
Hematocrit (%)	-0,025	0,839
White blood count (x10 [9]/l)	0,086	0,481
Iron(mcg/dl)	0,247	0,041^∗^
Total iron binding capacity (mcg/dl)	0,149	0,222
Transferrin (mg/dl)	0,177	0,146
Ferritin (ng/ml)	-0,129	0,289
Albumin (gr/dl)	0,164	0,178
Ca (mg/dl)	-0,118	0,335
P (mg/dl)	-0,092	0,452
CaxP	-0,084	0,493
	-0,081	0,511
IPTH (pg/ml)	0,046	0,708
Urea (mg/dl)	-0,020	0,870
Creatinine (mg/dl)	0,036	0,768
Uric acid (mg/dl)	-0,089	0,466
Total cholesterol (mg/dl)	-0,025	0,841
Triglyceride (mg/dl)	0,039	0,751
LDL (mg/dl)	0,021	0,863
HDL (mg/dl)	0,064	0,600
CRP (mg/dl)	-0,071	0,561
Kt/V	-0,001	0,997
Residual GFR (ml/mn)	0,142	0,262
Residual urine(ml)	0,086	0,489
D/P creatinine	-0,140	0,269

r: Spearman's correlation coefficient. ^∗^*p* < 0, 05. LDL: Low density lipoprotein; HDL: High-density lipoprotein; CRP: C-reactive protein; Kt/V: Clearance of urea normalized to total body water; GFR: Glomerular filtration rate; D/P: Dialysate/Plasma.

## Data Availability

The data that support the findings of this study are available from the corresponding author upon reasonable request.
